# Pitting of malaria parasites and spherocyte formation

**DOI:** 10.1186/1475-2875-5-64

**Published:** 2006-07-31

**Authors:** Samuel B Anyona, Stanley L Schrier, Charity W Gichuki, John N Waitumbi

**Affiliations:** 1 School of Pure and Applied Science, Department of Biochemistry and Biotechnology, Kenyatta University, Nairobi, Kenya; 2 Division of Haematology, Stanford University School of Medicine, Stanford, USA; 3 US Army Medical Research Unit-Kenya, and Kenya Medical Research Institute, Nairobi, Kenya

## Abstract

**Background:**

A high prevalence of spherocytes was detected in blood smears of children enrolled in a case control study conducted in the malaria holoendemic Lake Victoria basin. It was speculated that the spherocytes reflect intraerythrocytic removal of malarial parasites with a concurrent removal of RBC membrane through a process analogous to pitting of intraerythrocytic inclusion bodies. Pitting and re-circulation of RBCs devoid of malaria parasites could be a host mechanism for parasite clearance while minimizing the anaemia that would occur were the entire parasitized RBC removed. The prior demonstration of RBCs containing ring-infected erythrocyte surface antigen (pf 155 or RESA) but no intracellular parasites, support the idea of pitting.

**Methods:**

An *in vitro *model was developed to examine the phenomenon of pitting and spherocyte formation in *Plasmodium falciparum *infected RBCs (iRBC) co-incubated with human macrophages. *In vivo *application of this model was evaluated using blood specimens from patients attending Kisumu Ditrict Hospital. RBCs were probed with anti-RESA monoclonal antibody and a DNA stain (propidium iodide). Flow cytometry and fluorescent microscopy was used to compare RBCs containing both the antigen and the parasites to those that were only RESA positive.

**Results:**

Co-incubation of iRBC and tumor necrosis factor-alpha activated macrophages led to pitting (14% ± 1.31% macrophages with engulfed trophozoites) as opposed to erythrophagocytosis (5.33% ± 0.95%) (P < 0.01). Following the interaction, 26.9% ± 8.1% of the RBCs were spherocytes as determined by flow cytometric reduction in eosin-5-maleimide binding which detects RBC membrane band 3. The median of patient RBCs with pitted parasites (RESA+, PI-) was more than 3 times (95,275/μL) that of RESA+, PI+ RBCs (28,365/μL) (P < 0.01). RBCs with pitted parasites showed other morphological abnormalities, including spherocyte formation.

**Conclusion:**

It is proposed that in malaria holoendemic areas where prevalence of asexual stage parasites approaches 100% in children, RBCs with pitted parasites are re-circulated and pitting may produce spherocytes.

## Background

In the malaria holoendemic area of Lake Victoria basin, the estimated inoculation rate averages one infective bite daily and, as a consequence, prevalence rates of asexual stage malaria parasites in children is almost 100% [1]. The persistent parasitaemia induces profound haematological abnormalities that include anaemia, platelet depletion, cytoadherence of infected red cells, leukocytic stimulation by released parasite antigens, induction of cytokines and splenomegaly, among others [2]. In an attempt to contain these haematological aberrations, the macrophages of reticulo-endothelial system (RES) embark on clearance of parasites through mechanisms that are still not very clear [3].

Animal experiments conducted in late 60s suggested that the spleen can remove intraerythrocytic parasites while leaving the host erythrocyte intact, a process referred to as 'pitting' [4,5]. This process is analogous to splenic removal of intraerythrocytic inclusion particles such as Heinz and Howell-Jolly bodies [4,5]. Further evidence of pitting in patients with *Plasmodium falciparum *malaria [3,6] has recently come from demonstration of circulating RBCs containing abundant ring-infected erythrocyte surface antigen (pf 155 or RESA) but no intracellular parasites [3,6]. More recently, it has been demonstrated that, because of the rigidity that is associated with infected RBCs, trophozoites may be pitted out of infected erythrocyte by the shear pressure of the tight spleen capillary bed [7]. Whether by action of macrophages or capillary bed pressure, the product of pitting is creation of surface area depleted RBCs [8] that are free of parasites. Such RBCs are referred to as spherocytes, a term that indicate cells that are spheroidal (less disc like) than normal RBCs. Depending on proportion of RBC membrane that has been lost, spherocytes can have normal sizes or smaller in which case they are referred to as microspherocytes.

The growth of *P. falciparum *in the RBC leads to membrane insertion of parasite antigens such as RESA and *P. falciparum *erythrocyte membrane protein-1 (Pf EMP-1), and also induces profound modifications to the erythrocyte integral membrane proteins such as Band 3, which then becomes the sites for deposition of immunoglobulins or complement [9]. All these alterations of RBC surface are signals to macrophage attack and destroy these RBCs by erythrophagocytosis or perhaps lysis. Certainly the removal of large numbers of infected RBCs (iRBCs) and modified non-infected RBC is one of the mechanisms that contribute to development of malaria anaemia in children living in conditions of intense malaria transmission [10-12]. A competing mechanism of parasite clearance through the process of pitting with RBC salvage has been suggested as a host mechanism of attenuating anaemia [6]. The trigger mechanisms for RBC salvage as opposed to destruction are unknown.

This paper reports on high prevalence of spherocytes in children that are constantly exposed to malaria and provides evidence that this may be caused by pitting of malaria parasites. An *in vitro *model system of studying the process of pitting in an environment that is devoid of host factors is also described. This model may provide a method of identifying RBC membrane changes that trigger macrophages to engage iRBCs and, thereby, initiate the pitting process. The *in vivo *relevance of this model was evaluated using blood specimens from patients attending Kisumu Ditrict Hospital.

## Methods

### Patients samples

Giemsa-stained thin blood smears were made from patients enrolled in a case control study whose details have been described earlier [11,12]. Briefly, the case control study comprised three groups: cases of severe malarial anaemia (SA) defined as children with asexual *P. falciparum *parasitaemia and a haemoglobin level of ≤ 5 g/dL, and two controls, a symptomatic control (SC) and asymptomatic control (AC), each matched by age and gender to a case. SC were children with asexual *P. falciparum *parasitaemia and haemoglobin greater than 5 g/dL. AC were healthy children recruited from the same village as the cases. From each group, 25 thin blood films were examined by microscopy for abnormal RBC morphology. The haematologist slide's examiner had no prior information on the clinical status of the children. Blood specimens were also obtained from another group of 25 patients who were recruited in a case control study at Kisumu Ditrict Hospital. In this study, cases as defined above were matched with symptomatic controls of similar age and gender (± 2 months). In both studies, the protocols were approved by the Ethical Review Committee of the Kenya Medical Research Institute (KEMRI).

### Culture and synchronization of parasites

A laboratory strain of *P. falciparum*, NF54, was cultured in normal O^+ ^RBC from healthy donors and maintained *in vitro *using continuous culture conditions [13]. Parasites at an initial parasitaemia of 2% were enriched for ring stages using D-sorbitol [14]. Briefly, 6 mLs of culture at 2% parasitaemia of mainly young rings (10–12 h post invasion) were spun at 600 *g *and the pellet re-suspended in 6 mL 5% D-sorbitol. After 10 min incubation at room temperature, the cells were washed twice in malaria culture medium, diluted to 5% haematocrit and cultured as described above. This treatment was repeated every 48 hours until all the parasites were at ring stage as confirmed by microscopy. At 8% trophozoite parasitaemia, the iRBC were harvested by Percoll gradient purification method. Briefly, Percoll gradients were prepared by adding 10 mL of 90% Percoll to the bottom of a 45 mL Oakridge tube. Subsequent 5 mL layers of the 80%, 70% and 40% gradients were added consecutively. Parasites were suspended in 5 mL of RPMI 1640 and layered on top of the 40% gradient. This preparation was centrifuged in a SS34 fixed angle rotor (RC-5C Sorvall instruments, Du Pont, Maryland) at 10,000 rpm for 20 minutes, at 20°C. Trophozoite infected erythrocytes were removed from the second interphase and washed twice in RPMI 1640 containing 0.5% bovine serum albumin.

### Culture of monocytes

THP-1 cells were obtained from American Type Culture Collection (ATCC., Manassas, VA, USA) and were maintained in RPMI 1640 medium supplemented with 10% foetal bovine serum, 0.05 mM 2-mercaptoethanol, 100 U/mL penicillin, 100 μg/mL streptomycin, 1.0 mM sodium pyruvate, 2.5 g/L D (+) glucose and 2 g/L sodium bicarbonate at cell densities of 3 × 10^5 ^cells/mL in a humidified incubator at 37°C, 5% CO_2_.

### Co-incubation of iRBC and monocytes

Three triplicate THP-1 cultures were grown overnight (17–19 hours) in sterile 15 mL polypropylene tubes at a concentration of 3 × 10^6^/mL in 6 mL medium containing TNF-α at a final concentration 4 ng/mL. This treatment did not affect cell viability as determined by trypan blue exclusion. Activated monocytes were washed once in RPMI and co-incubated with 1 × 10^6 ^trophozoite iRBCs in a water bath for 1 hour at 37°C. The cells were washed twice by centrifugation and the resulting pellet resuspended in 80 μL of RPMI. Giemsa-stained thin smears were made using 10 μL of the cell suspension. Thereafter, the smears were examined by microscopy to identify: 1) pitted malaria parasites, 2) spherocytes and 3) the number of RBCs with and without the parasites.

### Eosin-5-maleimide (EMA) labeling of RBC

The EMA dye binds quantitatively to the RBC integral membrane protein Band 3 and can be used to reveal RBC membrane loss [15]. EMA preparation and storage was done as recommended by King et al [15]. Following co-incubation of iRBC and the monocytes, the cell mixture was washed, pelleted by centrifugation and resuspended in 50 μL of RPMI containing 0.5 mg/mL EMA (Eugene, Oregon USA). The procedure was used to stain patient RBCs. The mixture was incubated with intermittent mixing in the dark for one hour at room temperature. EMA stained cells were washed thrice and the pellet re-suspended in 500 μL of RPMI containing 0.5% bovine serum albumin. Acquisition and analysis were done immediately thereafter using a FACScan (Becton Dickinson, CA. USA) loaded with WinFCM and EXPO software (Applied Cytometry Systems, Sheffield UK). RBCs were gated on the basis of their logarithmic forward and side scatter characteristics. The level of EMA median logarithmic FL-1 channel fluorescence was determined for 15,000 events.

### Probing of patient RBC with anti-RESA monoclonal antibody and propidium iodide

For flow cytometry, patient RBCs (1 × 10^6^) were washed in PBS containing 1% BSA. The cells were then incubated with 1 mL 0.05% glutaldehyde for 30 minutes at room temperature. The glutaldehyde fixed cells were washed and permeabilized with 500 μL 1% Saponin in PBS for 5 minutes at room temperature. After two washes the cells were either resuspended in PBS containing 1 μg/mL RESA Mab, clone 28/2 (a gift from Robin F. Anders, Department of Biochemistry, La Trobe University, Bundoora, Victoria, Australia) and or in PBS containing 50 μg/mL propidium iodide and incubated at room temperature for 30 minutes. The cells were then washed twice with PBS and a FITC conjugated goat anti-mouse IgG-Fab fragment (Sigma-Aldrich Inc. St Luis, USA) added and cells incubated for 30 minutes at room temperature. RBCs were gated on the basis of their logarithmic forward and side scatter characteristics. 10,000 events were acquired and the proportions of RBCs that were RESA positive detected in the FL1 channel and those that were PI positive in the FL3 channel.

For fluorescent microscopy, thin blood smears were prepared and fixed with absolute ethanol for 3 minutes and air-dried. The slides were frozen at -20°C until used. Following removal from the freezer, the slides were washed gently in PBS and air dried for 5 minutes. The slides were then flooded with PBS containing either 1 μg/mL RESA Mab, and or 50 μg/mL propidium iodide and incubated for 30 minutes at room temperature. The slides were then washed twice with PBS and a FITC conjugated goat anti-mouse IgG-Fab fragment (Sigma-Aldrich Inc. St Luis, USA) added and the slides incubated as above. The slides were washed twice with PBS and examined using a BX40 fluorescent microscope (Olympus, Melville, NY), equipped with a Magnafire digital camera.

### Statistical analysis

Welch corrected t test was used to compare the number of monocytes with engulfed parasites against those with engulfed iRBCs from a set of six experiments. Results are given as mean and standard error of the mean (SEM). For detection of proportion of RBCs that were both RESA+ and PI- and those that were RESA+ and PI+, the % RBCs population identified by flow cytometry was multiplied by total RBC count in each sample as determined by Coulter. The Mann-Whitney test was then used to compare proportion of RBCs that were both RESA+ and PI- and those that were RESA+ and PI+. Dye binding results are expressed as median channel fluorescence (MCF) and SEM. The Kruskal-Wallis test was used to compare the dye binding results for the un-interacted iRBCs, interacted normal RBC and interacted iRBC.

## Results

### Spherocytosis and hypochromia is prevalent in all the clinical groups

As shown in Table 1, there was a high prevalence of spherocytes and hypochromia (reduced hemoglobin content) irrespective of the clinical status of the subjects. The higher number of patients with spherocytes in the SA group was statistically significant from AC group (two sided p value for SA vs AC = 0.04, Fishers Exact Test), but not significant from SC group (two sided p value for SA vs SC = 0.25, Fishers Exact Test). Out of the 75 slides examined, 73 were evaluable. 59 of the 73 had parasitaemia (81%), including half of the children in the AC group. 58 of the 73 had spherocytes (79%) and 49 of the 59 with parasitaemia had spherocytes (83%). 10/73 had spherocytes but no parasites (14%). 6/73 had neither spherocytes nor parasites (8%). Virtually all of the slides showed hypochromia and microcytosis (small RBCs with reduced haemoglobin content), indicative of significant iron deficiency. This is consistent with low plasma ferritin values that we observed in children without acute malaria [12].

**Table 1:** Number of patients with spherocytes, hypochromia and parasitaemia in the three clinical groups. Details of these patients have been described earlier [11-12]

	Severe anaemia N = 25	Symptomatic controls N = 24	Asymptomatic controls N = 24
Spherocytes	23 (92%)	19 (79.1%)	16 (66.7%)
Hypochromia	25 (100%)	24 (100%)	24 (100%)
Parasitaemia	25 (100%)	22 (91.7%)	12 (50%)

### Optimization of conditions for pitting

The success of pitting experiment was dependent on the ability to: 1) identify pitted malaria parasites from intact iRBCs co-incubated with the monocytes, and 2) detect increase in the number of uninfected RBCs as a result of this interaction. This required having a stage of malaria parasite that does not rupture during experimental manipulation and having a near 100% purified iRBCs before the interaction. The preferred method of preparing different stages of malaria parasites is by Percoll gradient. It was technically impossible to separate non-infected RBCs from ring stage iRBCs. But, while it was possible to obtain pure preparation of schizonts iRBCs by Percoll, during the 1 hour co-incubation with monocytes, many were found to have ruptured and it was, therefore, difficult to tell whether the engulfed schizonts were extracted from iRBCs or were from already ruptured parasites. Pure preparation of trophozoites iRBCs was easy to obtain by Percoll separation and they remained intact during the period of interaction with monocytes as confirmed by light microscopy. Therefore, all the experiments were conducted using trophozoites iRBCs.

**Figure 1:**
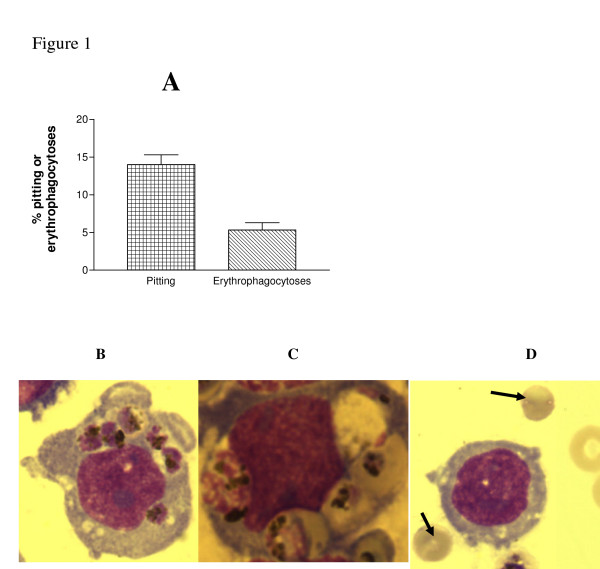
**Extraction of *P. falciparum *and pitting of infected red cells by THP-1 monocytes**. Trophozoite infected human group O^+ ^red cells (1 × 10^6^) in RPMI 1640 medium were co-incubated with TNF-α activated THP-1 monocytes (3 × 10^6^) for 1 hour at 37°C. Giemsa stained thin smears of the cultures were prepared and examined under oil immersion using ×100/1.3 objective of an Olympus microscope (Olympus America, NY). Pitting was preferred to erythrophagocytosis (panel A). The photomicrographs show monocytes with six pitted malaria parasites (B), phagocytosed infected RBCs (C) and spherocytic RBCs (D, arrow). Photomicrographs were captured using an Olympus MagnaFire camera (Olympus America, NY) using MagnaFire acquisition software.

For some reason, pitting was optimal when trophozoite iRBCs and monocytes were used at a ratio of 1:3. After 1 hour of interaction at this ratio, 14.0% ± 1.31% of the monocytes (mean of six experiments) were noted to contain malaria parasites in their cytoplasm as opposed to only 5.33% ± 0.95% found to have engulfed whole iRBCs (P < 0.01, Figure 1A).

The average number of pitted or erythrophagocytosed iRBCs was one, although multiple pitting and erythrophagocytosis were occasionally detected (Figure 1B and 1C, respectively). The number of RBCs devoid of trophozoites before and after interaction with the monocytes were enumerated. A 5% ± 1.5% increase in the number of un-parasitized RBCs was detected following the interaction (results not shown). The disparity between the reduction in parasitaemia and the number of monocytes with ingested naked parasites is probably due to lysis of damaged RBCs following the pitting process. RBCs devoid of parasites were smaller, globular and lacked a central hollow (Figure 1D) that is typical of normal human RBC.

### Detection of spherocytes by flow cytometry

**Figure 2:**
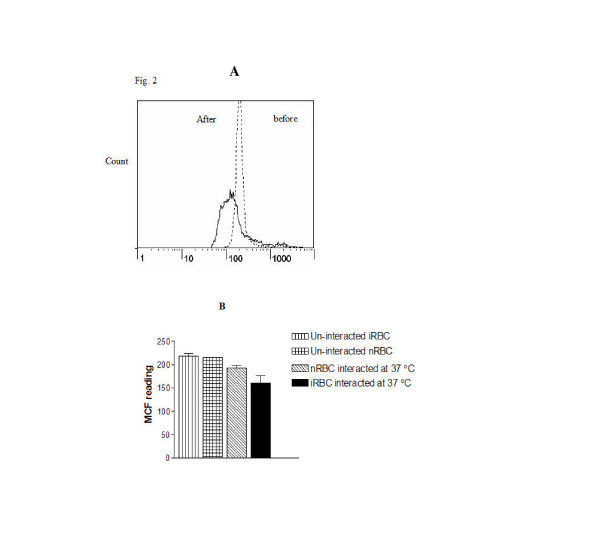
**Flow cytometric analysis of eosin-5-maleimide (EMA)-labelled *P. falciparum *infected (iRBC) and unifected RBC (nRBC) before and after interactions with THP-1 monocytes**. Trophozoite infected human group O^+ ^red cells (1 × 10^6^) in RPMI 1640 medium were co-incubated with TNF-α activated THP-1 monocytes (3 × 10^6^) for 1 hour at 37°C. Following the interaction, the cell mixture was stained with EMA by incubation in the dark for one hour at room temperature. Panel A is an overlay histogram of fluorescent profile of EMA labeled iRBC before (dashed lines) and after interaction (continuous lines) for the best of the 6 experiments. Panel B is a bar graph of median channel fluorescence (MCF, ± SEM) for 6 experiments of iRBC and nRBC following the indicated treatments.

Formation of spherocytes was studied by comparing the fluorescent intensity of iRBCs stained with EMA before and after co-incubation with monocytes. Following the co-incubation, the median channel fluorescence of co-incubated iRBCs was 26.9% ± 8.1% (P < 0.01) lower than that of un-incubated iRBC (Figure 2A and 2B). Co-incubation of uninfected RBCs led to a 10.1% decline in MCF but this did not reach significant level. Monocytes did not stain with EMA at all (results not shown).

EMA staining did not reveal presence of spherocytes in the RBCs of the 25 patients attending Kisumu District Hospital (results not shown).

### The proportion of parasite with pitted RBCs is higher than RBCs with parasites

**Figure 3:**
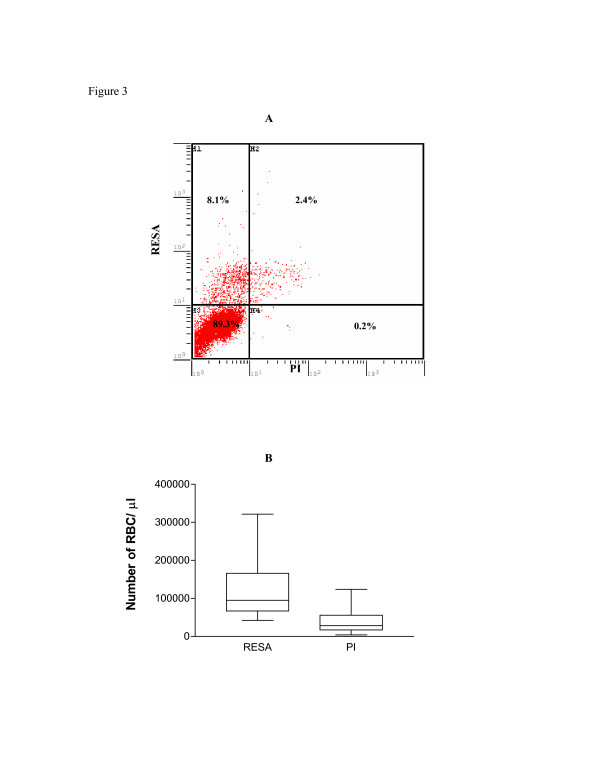
**Flow cytometric analysis of patient RBCs labeled with anti-RESA Mab and propidium iodide**. Patient RBCs were fixed with 0.05% glutaldehyde for 30 min prior to permeabilization with 1% Saponin for 5 minutes at room temperature. After two washes, the cells were stained with either RESA Mab, propidium iodide or both. The cells were then washed twice with PBS and a FITC conjugated goat anti-mouse IgG-Fab fragment added and cells incubated for 30 minutes at room temperature. (*A*) Flow cytometry dot plots of RBC labeled with FITC-anti-RESA (FL1-positive) and propidium iodide (FL-3 positive) showing proportion of RBC with pitted cells (RESA+, PI-) and those with malaria parasites (RESA+, PI+). (*B*) Box plotting demonstrate higher proportions of the once infected RBCs (RESA+) compared to those with malaria parasites (PI+).

**Figure 4:**
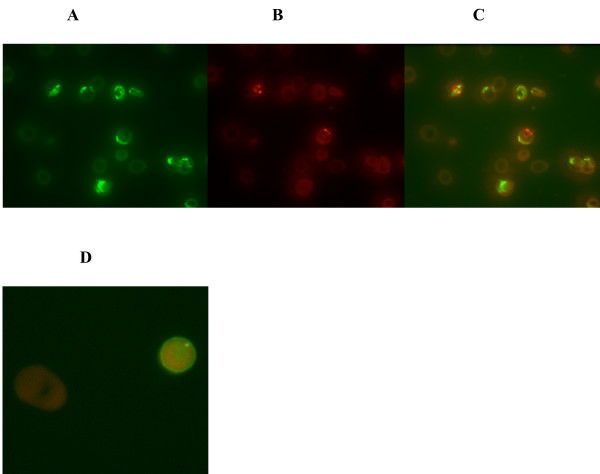
**Fluorescence microscopy images illustrating RESA and propidium iodide staining of patient RBC**. Thin blood smears were fixed with methanol and stained with either RESA Mab, propidium iodide or both. Images of the same field are shown in sets of three: a) green, b) red, and c) red + green composite. The number of RBCs showing pitted parasites (RESA+, panel A) were more than those with patent parasitaemia (PI+, panel B) and panel C. RBCs with pitted parasites showed other morphological abnormalities, including spherocyte formation (D).

Patient RBCs were probed for "footprint" of previous parasitization using anti-RESA Mab while patent infection was detected by propidium iodide (Figures 3 and 4). Using flow cytometry, the proportion of previously parasitized RBCs (RESA+, PI-) was always higher (95,275/μL) than that of RBCs with patent infection (28,365/μL) (RESA+, PI+, Figure 3A and 3B), and this was statistically significant (P < 0.01). Thin blood smears examined for RESA showed positive staining for ring infected RBCs as well as for the once infected RBCs (Figure 4). The number of RBC with previous infection (panel A) were more than those with patent parasitaemia (panels B and C), thus confirming the results obtained by flow cytometry. RBCs with pitted parasites showed other morphological abnormalities, including spherocyte formation (panel D).

## Discussion

A retrospective examination of blood films made from children with malaria revealed high prevalence of spherocytes regardless of their clinical status or parasitaemia (Table 1). It is not clear what the genesis of such high prevalence of spherocytes (79%) is, but it was speculated that the spherocytes reflect the pitting of malaria parasites from infected RBCs with a con-current removal of RBC membrane. Other studies have reported the phenomenon of pitting [3,6,7,16-20] and was recently shown to happen when the spleen traps the parasitized RBCs and pits out the parasites [21]. The study reported here is the first attempt to link pitting and occurrence of spherocytes in malaria. Spherocyte formation could have been enhanced by presence of activated macrophages and splenic enlargement is one of the indicators of macrophage activation. Indeed, splenomegaly was greater in SA (mean 3.6 ± 1.8 cm) than controls (SC = 0.8 ± 1.3 cm, AC = 0.1 ± 0.5 cm, P < 0.01) [12]. These findings prompted experiments to determine whether spherocytes could have been created by pitting of malaria parasites.

Model human monocytes (THP-1) were used to demonstrate removal of intra-erythrocytic malaria parasites (Figure 1). Pitting of trophozoites (Figure 1) was associated with loss of RBC membrane, as indicated by reduction of EMA binding (Figure 2). The EMA binding is known to quantitatively detect the RBC integral membrane protein, Band 3, and the reduction of Band 3 has been used as a screening test for hereditary spherocytosis [15,17]. It could be argued that the change in EMA staining that was observed after interaction with monocytes is due to dye loss from the interaction of RBCs with the monocytes with no real changes in membrane area. Indeed, some changes in EMA staining occurred when uninfected RBC were interacted with monocytes (Figure 2, panel B) but, the change in EMA staining was always much more pronounced when the interaction was carried with infected RBCs.

In an *in vitro *study, Kumaratilake *et al *interacted human peripheral leukocytes with cultured *P. falciparum *and showed that extraction of intraerythrocytic malarial parasites was preferred to ingestion of infected erythrocytes [16]. However, they did not comment on the formation of spherocytes after the parasites had been removed. In the current study, RBCs from patients were stained with EMA but the presence of spherocytes could not be revealed by this method. When interpreting this data, it is important to realize that some spherocytes have normal sizes and EMA will only reveal microspherocytes that result from removal of considerable proportion of RBC surface. In malaria, it is the circulating ring stage parasites that are pitted, unlike in the *in vitro *set up where macrophages were interacted with the much bigger trophozoite stages. It is conceivable that unlike in the trophozoite stages, pitting of the small ring stages would not involve removal of sizeable RBC membrane. Another explanation is that EMA is not sensitive enough to detect the small numbers of spherocytic cells that emanate from pitting. Fluorescent microscopy did however identify RESA+, *P. falciparum *negative RBCs that were spherocytic (Figure 4D). EMA would work best in situations whereby large numbers of spherocytes are created for example in hereditary spherocytosis [15,17].

The growth of *P. falciparum *induces profound modifications to the erythrocyte membrane. It causes oxidative damage to proteins such as Band 3 while intraerythrocytic parasite growth causes insertion into the membrane of antigens such as RESA and Pf EMP 1 [6,9,20,22]. *In vivo*, these alterations, particularly the haemichrome-mediated oxidation of Band 3, are thought to mark the iRBCs for immunoglobulin and complement deposition and subsequent erythrophagocytosis [22]. Although pitting was the dominant phenomenon in the *in vitro *model, erythrophagocytosis also occurred (Figure 1B) indicating that host factors do not have to be present for erythrophagocytosis to take place. Crucial to either pitting or erythrophagocytosis was activation of the THP-1 monocytes by TNF-α. Unstimulated monocytes did not engage in either of the two processes (results not shown). TNF-α was used to activate the monocytes because it is a major pro-inflammatory cytokine produced by macrophages of RES in children with malaria [23].

Patient RBCs were probed for "footprint" of previous parasitization using anti-RESA Mab. The proportion of previously parasitized RBC was consistently higher than that of RBCs with patent infection as determined by flow-ctytometry (Figure 3) and immunofluorescence (Figure 4). This is consistent with the estimated survival period for RESA positive, *P. falciparum *negative RBCs that are longer than that of parasitized RBCs [6].

In conclusion, *in vitro *data from this study indicate that pitting of malaria parasites can produce spherocytes. Taken together with other studies that indicate that pitting of malaria parasites is common [18,19], and indeed preferred to phagocytosis of infected RBC [16], it is proposed that, in malaria holoendemic areas where the prevalence of asexual stage malaria parasites approaches 100% in children [1], the day-in-day-out pitting of malaria parasites produces high spherocytic rates. Of course, spherocyte formation may be a consequence of the pitting of intraerythrocytic inclusion bodies like Howell-Jolly bodies or the Heinz bodies produced in conditions associated with RBC oxidant stress such as glucose-6-phosphate dehydrogenase (G6PD) deficiency. G6PD deficiency is common in malaria endemic areas [24]. It could be argued that pitting of parasites and returning surface area depleted RBC to the circulation is biologically preferable to erythrophagocytosis. Erythrophagocytosis results in removal of the entire iRBC and, thus, contributes to the clinically important anaemia. In contrast, pitting restores a red cell, albeit damaged, to the circulation. The damage is complex consisting of a loss of surface area and produces a spherocyte that can no longer undergo the full elliptical deformation required to traverse the microcirculation [25]. The neoantigens present on the damaged RBC membrane lead to deposition of immunoglobulins and complement, all of which are signals for macrophagic attack. Nevertheless, despite its relatively shortened survival, the damaged red cell may perform useful oxygen carrying functions.

Finally, the *in vitro *model described here may prove useful in dissecting out those RBC membrane changes that trigger macrophages to engage iRBCs and thereby initiate the pitting or erythrophagocytosis process.

## Authors' contributions

JNW designed the research, analysed the data and wrote the paper. SLS examined and interpreted the blood smears and helped write the paper. SBA performed the research. CWG helped in study design and writing of the paper.
